# Organisational responses to mitigate the effects of COVID-19 on healthcare workers: a qualitative case study from Bogotá, Colombia

**DOI:** 10.1186/s12913-021-06825-2

**Published:** 2021-08-11

**Authors:** Simon Turner, Natalia Niño, Carolina Segura, Natalia Botero-Tovar

**Affiliations:** School of Management, University of los Andes, Bogotá, Colombia

**Keywords:** COVID-19, Coronavirus, Colombia, Qualitative, Workforce, Human resources, Wellbeing, Coordination, Health system

## Abstract

**Background:**

Healthcare organisations have undergone organisational change to respond to COVID-19. This pandemic has presented challenges for employee adjustment, with impacts on the availability and coordination of human resources in healthcare. This study aimed to characterise the organisational actions regarding the coordination of human resources in healthcare within Bogotá, Colombia, to respond to the COVID-19 pandemic.

**Methods:**

We followed a case study approach to understand the response to the emergency taking into account the narratives of managerial actors who have been directly involved in the planning of guidelines oriented to face the pandemic or in the implementation of health services for COVID-19. Twenty-two interviews with multiple health system organisations within Bogotá were conducted between May and September 2020 and analysed thematically.

**Results:**

Three themes emerged from the analysis of the interview data: to retain human resources, to implement actions to improve the mental and physical health of the healthcare workers, and to enhance healthcare workers knowledge, skills and availability to respond to COVID-19.

**Conclusions:**

Organisational actions led by hospital managers to retain, protect, and train human health resources in the dynamic context of the COVID-19 pandemic were identified. Other system-wide organisations like scientific associations contributed to the coordination of human resources across hospitals to respond to COVID-19 in Bogotá, Colombia. The actions of hospital managers, and roles of system-wide intermediary organisations, in coordinating human resources need to be explored in other health system contexts facing COVID-19.

## Background

Human resources, one of the major inputs of health care systems, refer to “the different kinds of clinical and non-clinical staff responsible for public and individual health intervention” [1]. The COVID-19 pandemic has necessitated rapid, responsive planning of health services to meet both growing and new types of demand. Such changes have implications for human resources, including their allocation to new roles (e.g. transfer from elective to emergency care), and affected the experiences of the healthcare workforce due to uncertainty about the risks associated with an unknown virus, the implementation of new protocols, changes in working conditions, and the widespread need for personal protection equipment (PPE) [2, 3]. For instance, one study conducted in three Latin American countries, including Colombia, reported that the majority of healthcare professionals lacked access to resources such as PPE to respond to COVID-19 [4].

Much of the literature covering the impact of COVID-19 on healthcare human resources has covered the perspectives and needs of health professionals providing frontline care [3, 5–9]. However, the roles and experiences of healthcare managers in coordinating organisational responses to COVID-19 remain underexplored [10, 11]. Healthcare managers are not only affected by the pandemic; they are key actors in the planning and implementation of organisational activities that affect frontline workers and other human resources. Using the narratives of hospital managers, and representatives of scientific and professional associations, the objective of this paper was to describe and analyse the organisational actions relating to hospitals in Bogotá, Colombia, that aimed to mitigate the effects of COVID-19 on healthcare workers.

The findings presented cover the first six months of the COVID-19 outbreak in Colombia (March-September 2020). Colombia was part of the ‘third wave’ of countries that were hit by the virus after Asian and European nations. This paper aims to contribute to the literature on health system responses to COVID-19 in Latin American contexts that faced the emergency once Asian and European countries had documented some epidemiological and clinical evidence about the virus and the disease. Table 1 summarises key elements of Colombia’s health system.

**Table 1:** Colombia’s health system response

**Country characteristics**
∙ Colombia is an upper middle income economy with a mixed health system (at all levels of health care) that provides both public and privately-funded care [12]. ∙ Bogotá, the capital city of 7.4 million people, has experienced the majority of cases (approximately 30 %) of the country throughout the epidemic [13]. It has one point five general practitioners and 0.4 specialists for every 1,000 inhabitants, and a total of 3,428 Internists which is the speciality that is most required to face the emergency [14].
**Policy responses to COVID-19**
∙ In March 2020, the government declared COVID 19 as an economic, social and ecological Emergency. ∙ In March 2020, the government authorised the early graduation of doctors-in-training to increase the resource of professionals. ∙ In May 2020, the government declared COVID-19 infections in health professionals as an occupational disease and allocated financial resources to respond.

## Methods

### Study design and participants

This paper is derived from an ongoing qualitative study that is analysing how the Colombian health system responded to COVID-19 [15]. The methodology we followed was a case study approach [16] in order to understand the response to the emergency taking into account the narratives of the actors who have been directly involved in system planning to face the pandemic and the implementation of health services for patients with COVID-19. Case studies emphasise the relevance of the particular context of the organisations and their healthcare teams [17], in understanding the response to the pandemic. The Consolidated criteria for reporting qualitative studies (COREQ) checklist was used as a guide to describe important domains concerning the research team, study designs, analysis, and findings [18].

### Procedures

Twenty-two semi-structured interviews were conducted between June and September 2020 in Bogotá, Colombia. Study participants, recruited through purposive and snowball sampling, were governmental bodies (e.g. ministries, institutes, and secretariats) (*n* = 2); hospitals (*n* = 10); and local scientific associations (*n* = 10) (Table 2). Purposive sampling was used to ensure that a range of organisations at different levels of the health system in Bogotá were approached for interview (including governmental and planning organisations, healthcare providers, and intermediary organisations like scientific associations). Snowball sampling involved asking interviewees if they were able to provide contact details for other interviewees that were relevant stakeholders in the response of Bogotá’s health system to COVID-19. A key characteristic of the sample is that we aimed to conduct interviews with senior representatives (e.g. director level or equivalent) of each organisation that participated in the study. Interviewees were approached by email, telephone, or instant messaging (WhatsApp). In total, 47 participants were approached for interview, representing a participation rate of 46.8 per cent; the main reasons for non-participation were the lack of a response to our requests for an interview or repeated postponement of interviews. No participants withdrew from the study once they had participated in an interview.

**Table. 2:** General characteristics of interviewees

Site ID	Role of the representative	Organisation	Months worked in the response to COVID-19 prior to interview
**Total** ***N*** ** = 22**			
SH-B-001	Service coordinator	Public hospital	3
SH-A-001	President	Representative of Scientific and Academic Associations	3
SH-A-002	Member	Representative of Scientific and Academic Associations	3
SH-B-004	Hospital manager	Private hospital	3
SH-B-005	Manager	Public hospital	4
SH-B-006	Service coordinator	Public hospital	4
SH-A-003	President	Representative of Scientific and Academic Associations	4
SH-A-008	Member	Representative of Scientific and Academic Associations	4
SH-B-008	Hospital manager	Private hospital	4
SH-A-011	Manager	Representative of Scientific and Academic Associations	4
SH-B-011 P-A	Hospital manager	Public hospital	5
SH-A-013	President	Representative of Scientific and Academic Associations	5
SH-B-013	President	Representative of Scientific and Academic Associations	5
SH-B-014	President	Representative of Scientific and Academic Associations	5
SH-A-029	President	Representative of Scientific and Academic Associations	6
SH-B-015	Hospital manager	Private hospital	5
SH-A-016	Vice President	Representative of Scientific and Academic Associations	5
SH-B-016	Hospital manager	Private hospital	5
SH-B-017	Service coordinator	Public hospital	5
SH-A-005	Member	Private hospital	4
SH-A-023	Manager	Government and policymakers	5
SH-A-025	Manager	Government and policymakers	5

Ethics approval for this research was received from the institutional review board at Universidad de los Andes. All participants provided written informed consent before the interview. Audio files were saved using a unique code, and all transcriptions were anonymised by replacing participants’ names with codes. Given that data collection took place while Bogotá was observing physical distancing measures, all interviews were conducted virtually using either telephone or virtual communication platforms (typically Microsoft Teams). Audio or visual recording were utilised to carry out the interviews. Interviews lasted 55 min on average. The interviews were informed by a topic guide that has been published previously as a supplementary file alongside the study protocol [15]; however, the questions were adapted to each interviewee’s context [19]. The topic guide was not piloted formally but it was reviewed by the team periodically and aspects of its use were adapted (e.g. considering additional verbal prompts to follow-up on the questions posed). Interviews were conducted by female researchers, NN (PhD), CS (MD), NB (MD), all of whom were employed as trained researchers on this study (all authors have previously undertaken formal research training in qualitative research methods). Only the participants and members of the research team were present at the interviews. Transcripts were not returned to study participants, nor did they provide feedback on the interviews. Repeat interviews were not carried out.

Relationships with participants were not established until commencement of the study. All interviewees were issued with a participant information sheet which explained the aim and objectives of the study and the purpose of the interview with regard to fulfilling those objectives. It was made clear to the interviewee that the interviewer was conducting the interview as part of a nationally funded research study and that fulfilling the goals of this study was the driving interest for performing the interview.

In addition to the interviews, the collection of legal documents and guidelines (*n* = 10) informed our understanding of the conditions in which health professionals were situated before and after the emergency was declared in Colombia.

### Data analysis

All the interviews were transcribed verbatim and coded using New NVivo software. An interdisciplinary research team trained in public health, medical anthropology and management collectively read the interviews and analysed the data thematically. All interviews were dual coded by more than one researcher. Themes were derived from the dataset and developed using both deductive and inductive analysis [20], meaning that our approach was informed by the particular focus and objectives of our study, while being open to novel ideas discovered through the narratives expressed in the empirical data. For example, we were surprised by the significant impact of COVID-19 on the physical and mental health of the workforce which was not a particular theme in our study protocol [15]. Deductively, the objectives of the wider study from which this paper is derived - and an interview topic guide that was developed response to those objectives [15] - informed the types of themes explored in the interviews. Inductively, we first analysed the dataset by exploring how each participant described the particular experience of their organisation facing COVID-19. Second, we coded for organisational responses to COVID-19 drawing on ideas and examples expressed in the interview data. Having developed and analysed 32 codes on organisational responses to COVID-19, we focused on data classified under the code “human resources”. The coding tree related to this code contained actions, challenges, enablers, barriers and labour conditions of the health workforce involved in responding to COVID-19. Quotations are presented within the results to illustrate key ideas within each theme; all quotations are identified by an anonymised participant number.

## Results

The results are divided into three major themes: adaptations to retain human resources, addressing workers’ mental and physical health, and actions to maintain a trained workforce. The relationship between the major themes and the data relating to each theme, including minor or subthemes, is presented in Table 3.

**Table 3:** Interventions, adaptations, practices and actions to support health care workers

Theme category	Interventions, adaptations, actions and improvements	Type	
		Organisational level	Inter-organisational level
1. Organisational adaptations to retain human resources.	A. Implement joint efforts (employer-employees) to maintain jobs as: take holidays and negotiated temporary reducing the staff salary.	X	
	B. Relocate health workers from out-patient, programmed surgery and other ambulatory services.	X	
	C. Open and enhance homecare services and telemedicine.	X	
	D. Allow work from home.	X	
	E. Contract additional staff to respond to the high volume of patients.	X	
2. Actions to improve workers’ physical and mental health, foster motivation, and wellbeing	A. Implement on-site drills and biosafety protocols on the proper use of personal protection elements to mitigate the contagion.	X	
	B. Provide PPE according to their risk exposure.	X	X
	C. Implement in-house psychiatrists to accompany their emergency team as a response to mitigate fear.	X	
	D. Implement regular refreshment breaks and having collective prayers in COVID-19 areas to support teamwork between the frontline workforce.	X	
	E. Implement personalized transportation, develop technological tools and advocate to reduce violence and stigma against health workers.	X	X
3. Actions, and adaptations to enhance workforce knowledge, training, and availability	A. Develop partnerships and agreements to expand the availability of general practitioners and specialists in the country.		X
	B. Enhance Adaptations and actions to enhance knowledge, skills and competencies related to the management of complex patients in ICU.		X
	C. Allow open access to already existing online training libraries.		X
	D. Adapt traditional health training.		X
	E. Give the students the option of postponing their practices or allow them to continue voluntarily in the hospitals.		X
	F. Provide personal protection equipment to students.		X
	G. Remove students from all high-risk areas of contagion.	X	X

### Retaining human resources

Managers stressed the organisational adaptations that hospitals had to implement in order to retain their human resources. These actions changed over the six months that we explored in our study. In general, during the early stage of the emergency (March to approximately May 2020), some hospitals closed their outpatient services in preparation to receive a high volume of COVID-19 patients. Opposed to what was expected, some hospitals reported having their facilities relatively empty during the first months after the emergency was declared in the country. Due to that, some of the regular hospital services programmed such as elective surgery, outpatient clinics, radiology among others were closed temporarily, having a negative financial impact in the hospitals.

Within a context of significant financial uncertainty, managers had to implement several strategies to maintain the hospital’s human resources while balancing this with the organisation’s sustainability. Managers highlighted the importance of protecting as many jobs as possible not only as a way of caring for their workforce but also because they foresaw all their trained and experienced personnel were going to be needed at some point during the emergency. Administrative personnel within closed services were asked to take holidays, and in one case, the hospital negotiated reduced staff salaries temporarily to maintain their contracts:

*“The clinic’s billing fell to 50 % in April. That is a terrible drama. In other words, imagine yourself with expenses of more than 150 %, and billing half (…) One of the first things we did was to send people on vacations. With some administrative staff and professionals in outpatient services, we agreed to reduce a percentage of their salary” (SH-B-015, hospital manager).*

Once hospitals began to experience a higher volume of COVID-19 patients (approximately June 2020 onwards), managers reported new challenges associated with human resource availability. During this second stage of the pandemic, the hospitals relied on all available personnel and in some cases, they had to contract new staff. Managers highlighted how workforce roles were made flexible, so they were available to attend to the emerging needs of the hospital. In some cases, managers asked some staff to be reallocated to homecare services and telemedicine, while in other cases, doctors and nurses working from home were asked to attend the hospital to care for non-COVID patients:

*We had to transfer human resources from the emergency centres that were underutilised, and strengthen and grow in services that were being demanded massively by the pandemic, such as home care services, telephone counselling services, teleconsultation services, which is what we call “extramural services”* (SH-A-005, hospital manager).

In summary, hospitals faced different challenges throughout the first six months of the pandemic. During the early stage of the emergency, hospitals faced a reduction in activity as fewer patients attended hospital services. This organisational context represented a barrier to keep the workforce of hospitals in post and potentially available in case they were needed. After the first two months, and when most of the hospital services were open, managers’ decisions were oriented to reallocating their personnel and adjust their roles according to the emerging needs of each hospital.

### Addressing workers’ physical and mental health

The interviews highlighted health care managers’ concerns about the impacts of COVID-19 on healthcare workers’ physical and mental health. A recurrent theme was the barrier faced by managers about the lack of PPE for staff during the preparation and early phase of the pandemic, and uncertainty over those responsible for its acquisition. Managers had to invest in higher volumes of PPE as never before. In the words of a manager, the organisation had:

“*the need for use and protection, with personal protection elements, defined by the Ministry, aligned by the Ministry, also led us to invest in human resources, personal protection elements that were not used before the pandemic were consumed [in such] volumes, because they are specific personal protection elements for a type of virus, which this was not seen before”* (SH-B-005, hospital manager).

Within hospitals, on-site drills and biosafety protocols were implemented on the proper use of PPE to mitigate the contagion that existed among health workers, which worsened the lack of available health personnel and make them more familiar with the routines for treating patients safely:

*“We started to do drills, patient drills, and that made us a little less afraid of people. Drills of taking off and putting on personal protection equipment, drills of intubation with the equipment, taking the equipment to that stressful part of the drills, I think that was very useful for us.”* (SH-B-017, Service coordinator).

In response to concerns about healthcare workers’ mental health (e.g. associated with fear and uncertainty), a representative of a hospital described the use of in-house psychiatrists to accompany their emergency team. Managers faced the barrier of health professionals afraid of being rejected and stigmatised by society. Having professionals working under these conditions was also presented as a challenge for managers as it made it difficult to motivate people to continue working. A hospital director declared that:

*It has been difficult to motivate the staff that feels this way, so they continue working, they stay. This is one of the key elements we have to prioritise in case there is another pandemic, that there is always emotional support and ways to support human resources’ wellbeing.”* (SH-A-006, service coordinator).

An emergency care manager stated that the wellbeing and mental health of healthcare workers in intensive care units and emergency care was supported by in-house psychiatry and psychology services such as Balint groups (regular meetings with a trained facilitator for debriefing).

Other measures to foster and enable staff motivation and wellbeing included establishing regular refreshment breaks or ‘hydration situations’ and having daily prayers which led to more emphasis on teamwork. A service coordinator stated that wrap-up meetings among the healthcare staff allowed the introduction of a new motto:

*“We started to do each other’s watch, the vigilance of the other and with the motto of ‘if you protect yourself, you protect me’”* (SH-B-017, service coordinator).

Interviewees expressed concerns about fear of contagion and death, confinement measures, economic difficulties and social factors that affected some members of the general population’s attitudes toward healthcare workers, generating stigma and violence towards health personnel as potential sources of contagion. In the words of an interviewee:

“*the reality overwhelmed the surveys because the reality, for example, in the attacks on doctors and health personnel went from being a survey to threats with flower crowns, with obituaries sent in a threatening way to workplaces, with threatening calls,”* (SH-A-003, president).

Thus, a manager of a hospital implemented personalised transportation within the city for healthcare staff to guarantee their safety:

*“we had to have buses as school routes because they began to attack our people, we only had physical aggression in one, but there began to be all those difficulties, so we set up transport routes, shared transport”* (SH-B-011, hospital manager).

Technological tools were developed to follow up and monitor working conditions and attacks on medical doctors. Professional associations had a critical role in this task for the surveillance of COVID-19 cases in the healthcare workforce and their working conditions. At the system-wide level, scientific associations advocated provision of legal support for the healthcare staff, to incorporate details for monitoring infected healthcare workers into the official records and launched an app for the surveillance of attacks on medical personnel. In summary, the physical and mental health of healthcare workers were pivotal topics that emerged in the interviews with managers and scientific associations that faced COVID-19.

### Workforce knowledge, training, and availability

Participants stressed that the Colombian health system had a shortage of specialists, general practitioners, and nurses, among other healthcare workers. In addition to this shortage of professionals, the COVID-19 pandemic caused a high inflow of patients due to the fact that the COVID-19 virus has a rapid speed of contagion, generating a new challenge: the need to have more human health resources available to care for these patients.

In response to this need, hospitals, governmental agencies, and universities collaborated and developed agreements to expand the availability of general practitioners and specialists in the country by graduating both general practitioners and residents early, increasing workforce capacity:

*“Last week in my program six but the country graduated about 25 in total, even so, there are very few intensivists, each intensive care unit should have an intensivist, that is, there are not 7,000 intensivists in Colombia. The availability increased to around 1,500 residents”* (SH B 013, president).

Scientific associations, hospitals and academic entities came together with the aim of improving knowledge and skills for the management of patients in Intensive Care Units (ICU). System-wide collaboration produced online training, continuing education programs (e.g. managing complex patients in ICU, mechanical ventilation, respiratory therapy) to systematise clinical knowledge on managing COVID-19 patients:

*“We would not have been able to do all this without the participation and commitment of scientific associations, universities and providers, who joined an initiative of continuous training […]. We, therefore, have some virtual courses that have a level of accompaniment and tutoring when technology and the time of the health team allow it.”* (SH-A-023, Manager).

Other academic institutions allowed open access to online training libraries through a national repository (500 to 600 free teaching activities from 55 medical schools in the country), facilitating access to training resources:

*“from the point of view of human resource training in health, so one of the first decisions we took was to ask all the medical faculties in the country to collect their virtual courses for us to make a national repository.”* (SH-B-013, President).

In summary, inter-organisational cooperation across the health system underpinned human resource planning, with the aim of enhancing existing health care workers’ knowledge and skills and increasing the availability of human resources to respond to the pandemic.

## Discussion and conclusion

Since COVID-19 emerged, qualitative research on its impact on the health workforce has focused predominantly on health professionals’ experiences on the frontline [3, 9, 11]. Research has focused attention on the individual experiences of healthcare staff treating COVID-19 patients in the early stages of the pandemic. Coping and self-care strategies, feelings of growth under pressure, and the simultaneous occurrence of positive and negative emotions of the health care workforce have been documented [21]. Psychological and/or psychiatric care provided to professionals in hospitals or other healthcare settings are highlighted as priorities for managers [5, 22]. Authors such as Halcomb et al. [23] have reported job security as a concern of the health personnel during the pandemic. Little is known about how the organisational context, including managerial decision-making, affects the human resources available to respond to COVID-19. For instance, the types of actions taken by hospital managers to retain the workforce [24] in the early stage of the pandemic, how those decisions were made, and their implications for service provision considering the financial restrictions faced by hospitals [21].

This paper adds to this area of research by focusing on how organisational level responses to COVID-19 affected human resource planning in hospitals in Bogotá from the perspective of managers within individual hospitals and representatives of nursing and medical associations at the health system level. The narratives of these actors and how they describe their experiences developing actions to protect the health workforce is scarce in the literature at the timing of writing. Our study makes three contributions to an organisational perspective on COVID-19.

First, it describes processes of responsive planning of health services by hospital managers to meet growing and new types of demand for services. Shifting roles and responsibilities of healthcare workers have been exposed in the literature from the perspective of the impact on frontline personnel [25, 26]. However, this study highlights that COVID-19 also affected the roles of hospital managers as they become more engaged in responsive planning. Organisational interventions that emerged from such planning had implications for human resources by shaping the retention, protection, and continuing training of healthcare workers. Hospital managers’ emergent roles included allocating staff to new roles and responsibilities (e.g. transfer from elective to emergency care), mitigating uncertainty among staff about the risks associated with an unknown virus, the implementation of new protocols and technologies, making changes in contractual working conditions, securing the availability of PPE, and participating in changes to health workers’ education [2, 3, 27, 28].

Second, it highlights the challenges faced, and actions taken, by hospital managers to mitigate the effects of responding to COVID-19 on the physical and mental health of healthcare workers. Hospital managers needed to address not only the physical and psychological safety of the workplace, but also the potential risks to staff outside the workplace linked to negative attitudes towards healthcare professionals.

Third, the important role of inter-organisational relationships at the local health system level for responding to COVID-19 was identified. For instance, scientific associations played a key role in the defence and surveillance of healthcare staff wellbeing, and the types of adaptations the managers undertook to respond. Staff training within provider organisations in Bogotá was supported by the development of partnerships between hospitals, universities, and scientific associations which produced system-wide online courses, continuing education programs, and shared repositories of training material.

With regard to existing research on health system change, the findings show a pace and approach to change not typically associated with the health care sector, which is often characterised as slow to change and subject to challenges concerning collaboration across professional and organisational boundaries [29–31]. By contrast, our findings suggested the importance of system-level coordination in response to COVID-19 to develop the capacity and training of the workforce and, at the provider level, concern among hospital managers to introduce organisational interventions to improve staff wellbeing. Our findings support the concept that COVID-19 gave “permission” to introduce innovations [32]; implementation of workforce change was underpinned by the coordinating role of system-wide actors and managerial focus on working conditions on the front-line of care delivery. The latter has been documented in qualitative research worldwide [33]. However, given that COVID-19 has represented a pressing and severe threat to life, and health system responses have been subject to intense public and media scrutiny, it is not clear whether these aspects of the motivating context of change associated with COVID-19 will be sustained over time or generalisable to other contexts of care.

### Policy and practice implications

The novel nature and widespread impact of COVID-19 has presented challenges for human resource planning and staff training. Presentation of the disease was uncertain and management guidelines were not available which made it necessary to develop training courses on topics related to pathophysiology of the disease and critical management of patients [28, 34]. In Europe [26], Asia [25] and North America [27], health systems have faced common challenges with regard to the process of training undergraduate and graduate students in nursing and medicine. Our findings in relation to Bogotá point to the importance of facilitating inter-organisational collaborations across the health system to strengthen human resources. Clinical training in response to the pandemic in Colombia was coordinated through joint planning between hospitals, universities and professional associations. To expand clinical capacity, a favourable measure was the early graduation of trainee doctors which was enabled by the Ministry of Education and implemented by hospitals and universities. These interventions correlate with similar measures that have been implemented in other countries to continue health workers’ academic training [27, 28].

Hospital managers played an important role in attending to the psychological impact of COVID-19 on healthcare workers, within and beyond the workplace. Existing research by Tomlin and collaborators propose a model about how health organisations and managers can respond to mitigate the psychological impacts on healthcare workers across the different stages of the pandemic [35]. For example, the importance of flexible work approaches, supportive leadership, regular debriefing, and communicating a clear plan for assessment of and capacity escalation of the response to further waves of COVID-19. Additionally, this study highlights the value of daily team-based activities to improve staff morale and preparedness for responding to COVID-19, which mitigates uncertainty around new workplace routines (e.g. using PPE and handling COVID-19 patients) and acknowledges the psychological impact on staff of responding to COVID-19.

### Study limitations

A limitation of this study was that interviewees gave their insights at different times within a six-month period following onset of the pandemic in Colombia. This might contribute to varying and incommensurate views in relation to changing conditions during the pandemic. Our results distinguish between ‘earlier’ and ‘later’ organisational responses to COVID-19 in Colombia concerning demand for healthcare services (i.e. before and after the anticipated “tsunami”, as one hospital manager put it). As the pandemic is likely to affect health services for the foreseeable future, researchers should specify the time period of their research and aim to capture shifting organisational responses to COVID-19 over time.

The study was undertaken in the upper-middle income country of Colombia, relying on interviews with health system actors in Bogotá, and may not be generalisable to other contexts. However, given the international spread of the pandemic, we have observed similarities between the organisational response in Colombia and other countries. For example, simulation training for staff has also been used by other countries such as Canada in different hospital settings with good results [36]. As in the United States, modifying shifts, hours and hospital attendance for residents and students was another of the measures successfully adopted in Bogotá [24–26]. Further research could examine, in other health system contexts, the shifting roles of healthcare managers in coordinating human resources, the influence of system-wide organisations including scientific and professional associations in supporting such coordination, and the particular challenges and risks faced by healthcare managers associated with the country context.

### Conclusions

This study highlights the impact of COVID-19 on the health care workforce, and the role of hospital managers and system-level actors in supporting workforce capacity, training and wellbeing in response to COVID-19 over the first six months of the pandemic in Bogotá, Colombia. It highlights how COVID-19 encouraged a pace and approach to health system change not typically associated with the health care sector; further research is needed to examine the sustainability of collaborative approaches to change associated with COVID-19 identified in this study (e.g. managerial engagement with front-line challenges and system-level coordination of workforce training and capacity building) and its relevance to other care contexts.

## Data Availability

The dataset supporting the conclusions of this article is included within the article.

## Abbreviations

SRQR: Consolidated Criteria for Reporting Qualitative Studies
ICU: Intensive Care Units
PPE: Personal Protection Equipment
